# Plant Essential Oils: Dual Action of Toxicity and Egg-Laying Inhibition on *Tetranychus urticae* (Acari: Tetranychidae), Unveiling Their Potential as Botanical Pesticides

**DOI:** 10.3390/plants13060763

**Published:** 2024-03-08

**Authors:** Yijing Zhu, Taoqi Wu, Qianyu Hu, Wenze He, Yushi Zheng, Yongjian Xie, Qiong Rao, Xunyue Liu

**Affiliations:** Key Lab for Biology of Crop Pathogens and Insect Pests and Their Ecological Regulation of Zhejiang Province, College of Advanced Agricultural Sciences, Zhejiang A & F University, Hangzhou 311300, China; yijingzhu@stu.zafu.edu.cn (Y.Z.); wtq0801@stu.zafu.edu.cn (T.W.); zhibao21qyhu@stu.zafu.edu.cn (Q.H.); hewenze@zafu.edu.cn (W.H.); yszheng@stu.zafu.edu.cn (Y.Z.); yjxie@zafu.edu.cn (Y.X.)

**Keywords:** plant essential oil, *Tetranychus urticae*, toxicity, egg-laying, botanical pesticide

## Abstract

*Tetranychus urticae*, a prominent pest mite in strawberry and vegetable cultivation in China, has developed escalating resistance due to extensive chemical pesticide application. Consequently, there is an urgent need to identify safe and efficacious methods to reduce resistance development. In this study, 38 commercially available plant essential oils (EOs) were screened for their acaricidal potential and ability to inhibit oviposition. The findings revealed that 13 EOs exhibited notable acaricidal activity, with lemon EO demonstrating the highest toxicity, followed by sage, patchouli, frankincense, lemongrass, palmarosa, and oregano EOs. In addition, 18 EOs displayed significant inhibitory effects on oviposition, with lemon EO exhibiting the highest inhibition rate (99.15%) and inhibition index (0.98). Subsequently, sage, frankincense, clove, lemongrass, oregano, patchouli, myrrh, black pepper, palmarosa, and geranium EOs also showed inhibition rates exceeding 50%. Despite black pepper, clove, myrrh, and oregano EOs demonstrating relatively low toxicity against *T. urticae*, they exhibited heightened efficacy in inhibiting oviposition and suppressing population expansion. This study conducted a comparative assessment of the acaricidal and oviposition inhibition activities of EOs and their principal constituents, thus providing a theoretical basis for the development of botanical acaricides against *T. urticae*.

## 1. Introduction

The two-spotted spider mite, *Tetranychus urticae* Koch (Arachnida: Tetranychidae), is extensively distributed across various countries worldwide, posing a significant threat to agriculture by causing severe damage [1]. *T. urticae* has a wide host range, encompassing over 1000 plant species from more than 250 families, including economically significant crops such as tomatoes, peppers, cucumbers, strawberries, apples, grapes, citrus fruits, corn, soybeans, and others. Its impact is particularly severe in greenhouse production [2,3]. The mite inflicts sap-sucking damage by puncturing leaf mesophyll cells and feeding on cell cytoplasm, which leads to extensive cell and tissue necrosis in plants, and may even result in leaf abscission, ultimately affecting crop quality and yield [4]. According to research statistics, nearly 80% of the total market value of acaricides, which were used to control spider mites, was accounted for, with only *Tetranychus* spp. (with *T. urticae* as the main species) representing EUR 372 million (62%) in 2008 [5].

Currently, the control of *T. urticae* still relies mainly on chemical pesticides. However, due to the repeated application of these chemical agents and the short lifecycle and strong reproductive ability of *T. urticae*, they can quickly evolve resistance to insecticides and acaricides within a short period of time, leading to an increasing difficulty in control [1,6,7,8]. According to the data from the arthropod pesticide resistance database, *T. urticae* has developed resistance to 96 active ingredients so far [9]. In light of the development of resistance in *T. urticae*, it is necessary to continue monitoring the selection and use of chemical agents, which is crucial. Moreover, it is important to seek environmentally friendly biopesticides whose modes of action can complement or replace traditional chemicals, aiming to slow down the development of resistance [10].

Plant essential oils (EOs) have rare adverse effects on the environment and human health compared to conventional synthetic pesticides [11]. The complex and diverse composition of botanical essential oils is a mixture of various active substances with different mechanisms of action. When used as insecticides and acaricides, they often achieve the purpose of insecticidal and acaricidal effects through multiple modes of action and mechanisms, making it difficult to develop resistance [12]. EOs, which can serve as fumigants, repellents, contact agents, feeding inhibitors, ovicidal agents, and oviposition deterrents against pests and mites [13], are typically employed through contact, ingestion, or inhalation to exert toxic effects by suppressing or altering key neurotransmitters and enzymatic and non-enzymatic antioxidant defense systems, including the modulation of the octopaminergic system and inhibition of the gene expression of *P450* cytochromes (*CYPs*), acetyl-cholinesterase (*AChE*), γ-aminobutyric acid receptor (*GABA*), superoxide dismutase (*SOD*), catalase (*CAT*), peroxidase (*Pox*), and glutathione-S-transferase (*GST*), among others [12,14]. 

There is research indicating that an intense toxicity towards *T. urticae* was displayed by fennel and lavender EOs in contact and fumigation bioassays [15]. The bioassay showed that the fumigation effect of gardenia EO against whitefly *Bemisia tabaci* adults was the best, with a mortality rate of 81.48%, and it also showed the best contact-killing effect on nymphs of *B. tabaci* [16]. 

The essential oils, which not only exhibit high toxicity against pests, but also affect their reproduction, have been observed in various studies. For example, squalene EO has been found to significantly inhibit the oviposition of whiteflies in greenhouse experiments [16]. Thyme EO has been shown to have anti-oviposition and anti-eclosion effects on bean weevils [17]. Stepanycheva et al. evaluated the toxicity and reproductive impact of 15 botanical EO products on thrips *Frankliniella occidentalis*, finding that all EOs significantly reduced the fecundity of adult females [18]. After spraying the wild tomato leaf ethanol extract on *T. urticae*, compared to the control, the average number of eggs laid per female mite decreased by 65% and 68% at 4 h and 24 h, respectively, indicating the inhibitory effect of the extract on oviposition [19].

*T. urticae*, characterized by its substantial resistance, poses a severe threat to a wide array of crops. It is imperative to explore environmentally and human-friendly control methods that are cost-effective. This study aimed to evaluate the acaricidal activity and oviposition inhibition activity of 38 commercially available EOs against *T. urticae* using the leaf-dipping bioassay method, providing a reference for the screening and utilizing EOs in the control of *T. urticae*.

## 2. Results

### 2.1. Preliminary Screening of Acaricidal Activity of EOs against T. urticae

**Figure 1:**
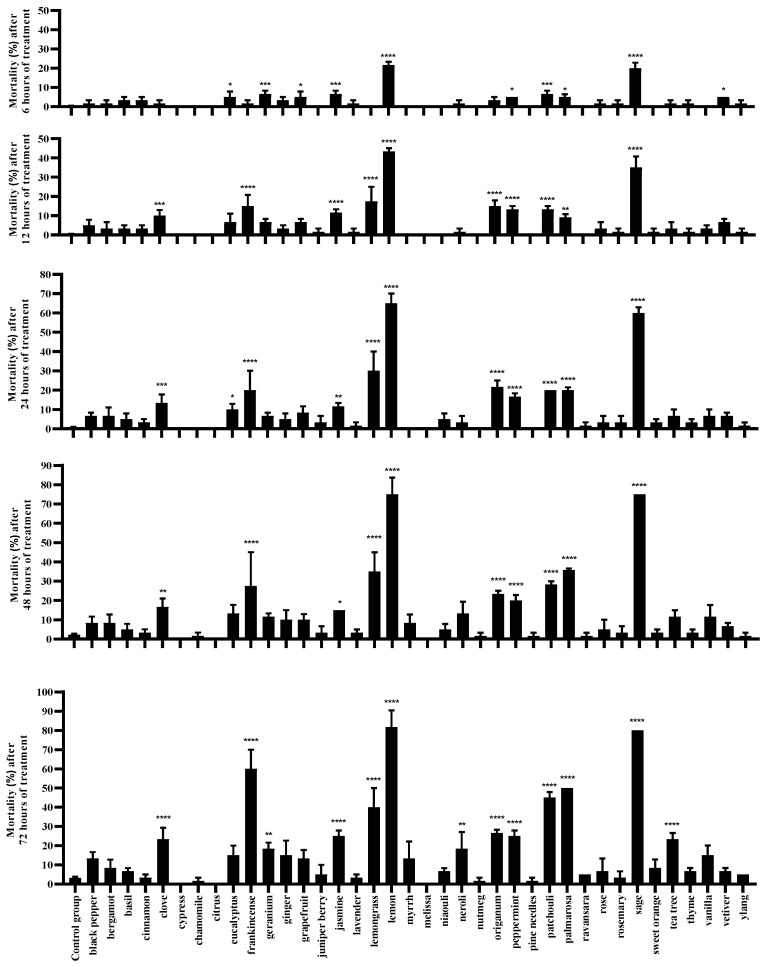
Mortality rates of *T. urticae* treated with plant essential oils at a concentration of 4 μL/mL at different time intervals (* *p* ≤ 0.05; ** *p* ≤ 0.01; *** *p* ≤ 0.001; **** *p* ≤ 0.0001).

After treating *T. urticae* with a leaf-dipping bioassay method using a concentration of 4 μL/mL of each of the 38 EOs, significant differences (*p* ≤ 0.01) in mortality rate compared to the control were observed 6 h after treatment for five EOs. Among them, lemon and sage EOs exhibited the highest acute toxicity with the most significant difference (*p* ≤ 0.0001) (Figure 1). The mortality rate increased with extended exposure time for most EO treatments. Specifically, at 12 h, the mortality rate significantly increased in the groups treated with frankincense, lemon, lemongrass, and sage EOs. By 24 h, frankincense, lemon, lemongrass, patchouli, peppermint, geranium, palmarosa, and sage EOs exhibited a certain level of toxicity, indicating an extremely significant difference compared to the control (*p* ≤ 0.0001). Additionally, at 48 h, the groups treated with lemon and sage EOs had the highest mortality rates, and by 72 h, a total of 13 EOs exhibited significantly higher mortality rates compared to the control group (*p* ≤ 0.01). Lemon EO demonstrated the highest toxicity against *T. urticae*, with an average mortality rate as high as 81.67%, followed by sage EO with an average mortality rate of 80%. Meanwhile, frankincense and palmarosa EOs displayed average mortality rates of 60% and 50%, respectively, while the remaining seven EOs showed mortality rates ranging from 20% to 50% (Figure 1).

### 2.2. Toxicity of Seven Selected EOs on T. urticae

The seven selected EOs were tested against *T. urticae*, and the results are presented in Table 1. At 6 h, lemon and sage EOs exhibited fast-acting effects. At 12 h, the LC_50_ values of some EOs significantly decreased, indicating a gradual increase in their toxicity to *T. urticae*. By 24 h, lemon EO had the lowest LC_50_ value, measuring only 3.539 μL/mL, which was significantly lower than those of the other EOs. Over time, at 48 h, all EOs’ LC_50_ values dropped below 10 μL/mL, and at 72 h, the LC_50_ values of lemon, patchouli, and sage EOs decreased to below 5 μL/mL. Based on their LC_50_ values after 72 h, the toxicity of the seven EOs against *T. urticae* was ranked. Lemon EO exhibited the highest toxicity with an LC_50_ value of 2.310 μL/mL, followed by sage, patchouli, and frankincense EOs, with LC_50_ values around 4 μL/mL. Lemongrass and palmarosa EOs had LC_50_ values of approximately 6 μL/mL, while oregano EO showed a lower toxicity, with an LC_50_ value of 9.123 μL/mL.

### 2.3. Effects of EOs on Oviposition of Female T. urticae

**Figure 2:**
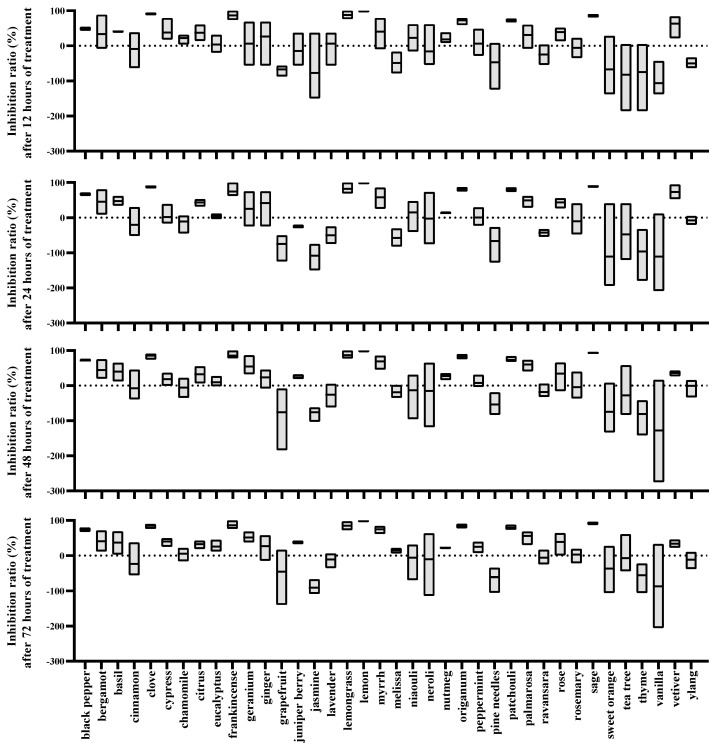
Oviposition inhibition rates of *T. urticae* treated with 4 μL/mL plant essential oils at different time intervals. (The dotted lines represent an inhibition rate of 0).

The study revealed that the EOs exhibit both inhibitory and stimulatory effects on the oviposition of female *T. urticae*, as shown by negative values (Figure 2 and Table 2). At 12 h, the inhibitory indices of clove, frankincense, lemongrass, lemon, patchouli, palmarosa, sage, and lemongrass EOs on the oviposition of female *T. urticae* exceeded 0.5, with inhibition rates surpassing 60% in all cases. Lemon EO had the highest inhibitory index of 1.00, corresponding to a 100% inhibition rate. Clove EO, despite exhibiting a lower inhibitory index of 0.59, still exhibited a high inhibition rate of 91.45%. By 24 h, the inhibitory index of black pepper EO rose to 0.51, with a corresponding inhibition rate of 67.50%. Although the inhibitory indices of frankincense and lemongrass EOs slightly decreased, they still demonstrated strong inhibitory effects on the oviposition of female *T. urticae*, with inhibition rates exceeding 70%. Over time, the inhibitory indices of most EOs either increased or fluctuated within a certain range. 

At 48 h, a total of 18 EOs were identified to inhibit the oviposition of female mites based on their inhibitory indices. Myrrh and palmarosa EOs exhibited increased inhibitory indices of 0.56 and 0.55, respectively, accompanied by inhibition rates of 69.35% and 59.83%. Additionally, the effect of juniper berry EO on the oviposition of female mites shifted from a stimulatory effect to an inhibitory effect, as evident from the rise in its inhibitory index from −0.11 to 0.13. Conversely, lemongrass EO showed a significant drop in its inhibitory index, reaching just 0.22, indicating a gradual decline in its impact on oviposition. 

At 72 h, the EOs were ranked in terms of their inhibition rates on egg-laying, which exceeded 0.00%. Lemon EO exhibited the highest inhibition rate (99.15%) and inhibitory index (0.98), followed by sage, frankincense, clove, lemongrass, patchouli, palmarosa, myrrh, black pepper, palmarosa, and geranium EOs, all surpassing a 50% inhibition rate. EOs such as grapefruit, basil, cypress, orange, juniper berry, rose, and lemongrass had comparatively weaker inhibitory effects, with average inhibition rates ranging between 30% and 50%. Furthermore, 12 EOs were found to promote the egg-laying of female mites, as indicated by negative average egg-laying inhibitory indices. Notably, jasmine, pine needle, and thyme EOs exhibited significant promoting effects, with inhibition rates of −90.97%, −60.72%, and −55.56%, respectively. The remaining EOs had both promoting and inhibitory effects on egg-laying.

**Figure 3:**
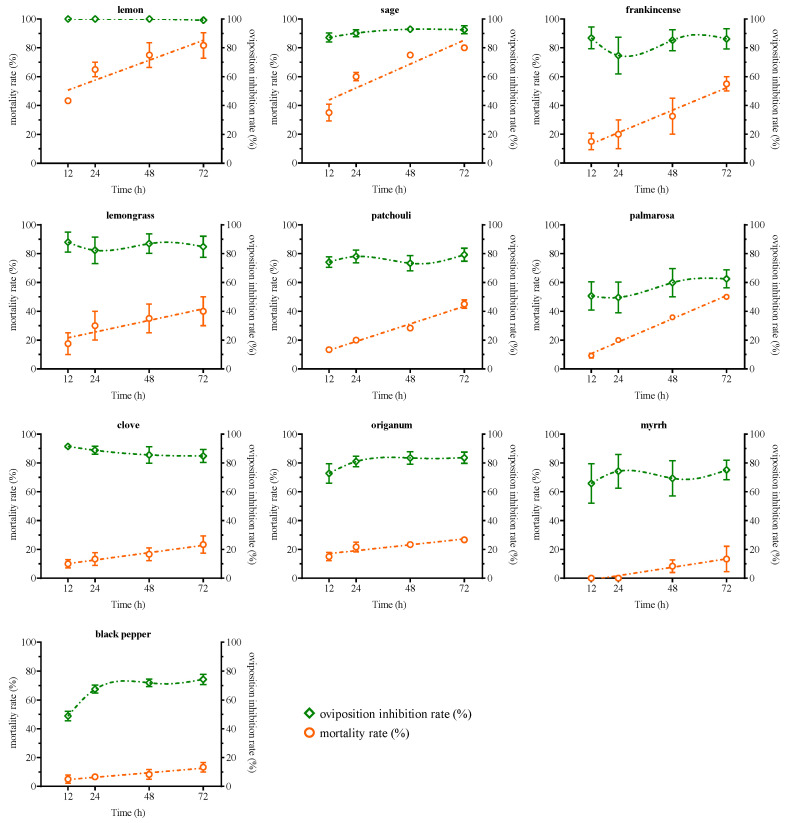
A comparison of mortality and oviposition inhibition rates of *T. urticae* treated with 10 different plant essential oils at a concentration of 4 μL/mL.

Frankincense, lemongrass, lemon, palmarosa, and sage EOs consistently maintained an oviposition inhibition index of 0.6 or higher for female *T. urticae*, with inhibition rates exceeding 70% (Table 2). At 72 h, *T. urticae* treated with lemon, sage, frankincense, lemongrass, patchouli, and palmarosa EOs showed average mortality rates exceeding 40%. Among them, excluding palmarosa, the other five EOs exhibited oviposition inhibition rates exceeding 80%, with lemon and sage EOs showing inhibition rates close to 100% (Figure 3). Although palmarosa achieved a mortality rate of over 40% on *T. urticae*, it had the lowest oviposition inhibition rate among the ten tested EOs. Conversely, black pepper, clove, myrrh, and oregano EOs exhibited lower mortality rates for *T. urticae* but demonstrated evident oviposition inhibition effects (Figure 3). Clove EO, which exhibited the strongest oviposition inhibitory effect on female *T. urticae*, maintained a rate consistently above 80% despite experiencing a slight decrease over time. The inhibitory rates of the other three EOs showed an upward trend from 12 h to 48 h, and then gradually stabilized from 48 h to 72 h in terms of oviposition inhibition rates. The oviposition inhibition rates of these three EOs were able to reach approximately 75% at 72 h (Figure 3). 

## 3. Discussion

Assessing the toxicity of EOs on different test subjects is a crucial aspect in evaluating their biological activity, and numerous studies have already demonstrated significant variations in the toxicity of different EOs on various tested pests. Based on the results of the bioassay tests in the study, it was observed that lemon and sage EOs demonstrated the most effective acaricidal effect on *T. urticae* after 72 h of treatment. Additionally, eucalyptus, frankincense, lemongrass, palmarosa, and oregano EOs also exhibited significant efficacy. Choi et al. [20] demonstrated that sage and lemongrass EOs exhibited high fumigant activity against *T. urticae* using the filter paper diffusion method. Lim et al. also found that lemongrass EO treatment through fumigation resulted in an average mortality rate of approximately 85.8% [21]. An et al. determined that lemongrass, sage, and eucalyptus EOs exhibit a higher level of toxicity against *T. urticae* [22]. These results, which are consistent with the conclusions of this study, demonstrate that these EOs not only have contact-killing effects on *T. urticae* but also exhibit excellent fumigant activity. In this study, frankincense EO exhibited higher toxicity towards *T. urticae* (with an LC_50_ value of 4.611 μL/mL) but a lower toxicity towards *Panonychus citri* McGregor (with an LC_50_ value of 21.953 μL/mL) [23]. On the other hand, patchouli and citrus EOs demonstrated higher toxicity towards *P. citri* [23], while showing lower toxicity towards *T. urticae* here. In addition to its effects on tetranychid, the study of EOs on other pests has also received attention. It has been found that lemon EO has potential lethal effects on Lepidoptera pests, and its modes of action include contact-killing, fumigation, repellency, and antifeedant properties. Lemon EO at a concentration of 2% exhibited a 34.2% antifeedant effect, 9.4% contact-killing effect, and 35.33% fumigation effect on the larvae of Lepidoptera pest *Mythimna separata* [24]. Additionally, lemon peel EO also showed certain insecticidal effects on the Lepidoptera pest *Agrotis ipsilon* [25]. Sage EO has certain fumigation and repellent effects on *Bemisia tabaci*, *Rhyzoperta dominica*, and *Ephestia kuehniella* [26]. Ylang ylang and frankincense EOs showed significant insecticidal activity against *Culex quinquefasciatus* larvae and *Musca domestica* adults [27]. Therefore, the same EOs may have toxic effects on multiple pests and mites, with variations in their specific toxicities. In the process of studying the insecticidal and acaricidal activity of EOs, it is necessary to comprehensively screen different EOs and select those within the appropriate concentration range based on the results. Furthermore, the toxic effects, including LC_50_ values and other data, should be compared to determine the efficacy of EOs in controlling different target pests. This provides a foundation for research on field control and pesticide formulation.

The evaluation indicators for the biological activity of EOs include, in addition to directly measuring their lethal toxicity to pests, the oviposition inhibition activity of the EOs, which can also serve as one of the indicators for evaluating their activity. The reduction in egg-laying has a direct impact on the decrease in the population size of insects. Relative to directly assessing the toxicity of EOs on *T. urticae*, studying their oviposition inhibitory activity indirectly contributes to evaluating the effectiveness of the EOs in pest control. It was found in this study that clove, frankincense, lemongrass, lemon, marjoram, palmarosa, and sage EOs exhibited significant inhibitory effects on female *T. urticae* oviposition at 12 h, 24 h, 48 h, and 72 h, with an inhibition rate exceeding 70%. The oviposition inhibition rate of black pepper, myrrh, and geranium EOs gradually increased with the extension of time, indicating that these EOs exhibit higher activity in inhibiting oviposition in adult female mites. Previous studies had shown that clove, holy basil, lemongrass, and fumigation achieved a mortality rate of up to 100% against the bean weevil *Callosobruchus chinensis*. Additionally, these fumigation methods exhibited excellent oviposition deterrent and inhibition of F_1_ offspring effects [28]. Sage EO exhibited an oviposition inhibition rate of 71.17% against the diamondback moth [29]. In addition, it was observed that some of the EOs, such as jasmine, pine needle, and thyme EOs, have the ability to stimulate oviposition in adult female mites.

Others studies have shown that both oregano and clove EOs can lead to a reduction in the number of eggs of *T. urticae* on legume plants and tomatoes. At a concentration of 0.25%, these two EOs decreased the egg production of *T. urticae* on the fifth day after treatment by approximately 1.60-fold and 1.68-fold, respectively [30]. Awad et al. compared the effects of clove, basil, and peppermint EOs on *T. urticae* and found that clove EO exhibited oviposition inhibition activity comparable to that of avermectin. On the other hand, the oviposition inhibition efficacy of basil and peppermint EOs was poorer [21]. This is consistent with the findings of our study, indicating that clove and oregano EOs can effectively inhibit the reproduction of mites, thereby reducing the population of the subsequent generation. In this experiment, sandalwood EO exhibited relatively weak oviposition inhibition, with only a 28.57% inhibition rate at 72 h. However, Roh et al. found that when 0.1% sandalwood EO was applied using the leaf-dipping bioassay method, it not only showed significant acaricidal activity against adult female *T. urticae* but also significantly inhibited their oviposition, resulting in an 89.3% reduction in the total number of eggs on the leaf disc [31]. This contradicts the findings of our experiment, suggesting that differences in the quantity of EO components may have an influence on its biological activity. 

The composition of EOs is complex, and further clarification of the active components against pests is needed to better understand their mode of action. Patchouli EO, along with its primary constituent, pogostone, has been observed to notably extend the developmental period of noctuid insects, specifically *Spodoptera litura* and *Spodoptera exigua*, encompassing both their larvae and pupae stages. Additionally, it has shown moderate ovicidal activity and impacts on the eclosion and deformities of these moths [32]. Oregano EO, along with its major components thymol and carvacrol, exhibit good potential for the control and prevention of the lepidopteran pest *Thaumetopoea wilkinsoni* [33]. The volatile compounds and polyphenolic compounds present in EOs might also be involved in the inhibition of acetylcholinesterase, thus affecting the nervous system of pests [34]. Clove EO and its principal constituent, eugenol, may serve as effective natural molluscicides against the land snail, *Theba pisana*, by influencing detoxifying enzymes. This indicates their potential in controlling and managing populations of this particular molluscan species [35]. Considering the potent biological activity demonstrated by certain components within EOs, subsequent research efforts can be focused on unraveling the chemical composition and mechanisms of action of these active EOs in this study.

In addition, there can be a synergistic enhancement effect observed in the combination of EOs with pesticides or other EOs. When 2% lemon EO was combined with the insecticide indoxacarb, a synergistic effect was observed [24]. Lemongrass and patchouli EOs exhibited little acaricidal activity against *Tetranychus cinnabarinus*, while a combination of pepper EO, mint EO, and avermectin demonstrated a significant synergistic enhancement effect [36]. Lemon EO exhibited a good repellent effect against the stored-product pest *Alphitobius diaperinus*, with an even stronger repellent effect observed when it was combined in a 1:1 ratio with lemongrass EO [37].

Research on the encapsulation materials of EOs is also crucial for the practical application of EOs in the field [38]. Lemon peel EO and its nanomaterials could enhance the insecticidal efficacy [25]. The utilization of nanoemulsion as an encapsulating material for basil, fennel, oregano, and chamomile EOs significantly enhances the insecticidal effect against *Aphis craccivora*, while also inhibiting the activity of insect detoxification enzymes [39]. The patchouli EO exhibited a good insecticidal effect against the larvae of *Culex pipiens* mosquitoes, with its nanoemulsion formulation further enhancing the efficacy by threefold [40]. The encapsulation of EOs through the formation of inclusion complexes (IC) using β-cyclodextrin (β-CD) achieved better insecticidal efficacy [26,41]. The encapsulation materials of these bioactive compounds not only enhanced their insecticidal efficacy but also promoted their protection against environmental degradation, thereby prolonging their bioactivity [42].

In addition to its insecticidal properties, EOs also exhibited simultaneous bactericidal and weed control effects. For instance, sage EO demonstrated antimicrobial activity against plant pathogens such as *Zymoseptoria tritici* and *Fusarium culmorum*, while also displaying significant anti-germination effects on lettuce and rye grass [26]. The research conducted by our research group has found that patchouli and Vetiver essential oils exhibit certain inhibitory effects against the citrus red mite, *P. citri* [23], as well as the citrus disease pathogen, *Penicillium digitatum* [43]. The screening of EO varieties that possess both antibacterial and insecticidal properties can be more time-efficient, labor-saving, and cost-effective. Due to the easy accessibility of many EOs in the flavor industry, they may further be considered as promising ingredients for use in plant formulations to combat pests.

From a simultaneous analysis of acaricidal activity and reproductive effects, this study found that frankincense, lemongrass, lemon, myrrh, oregano, pennyroyal, and sage EOs not only exhibited high toxicity against *T. urticae* but also inhibited its oviposition, demonstrating effective population control capabilities. Palmarosa EO, although demonstrating significant acaricidal activity, did not exhibit a noticeable effect on oviposition inhibition. However, black pepper, geranium, and fennel EOs, while not showing significant acaricidal activity at low concentrations, exhibited high oviposition inhibition indices and rates. Therefore, in the practical application of EOs for pest control, consideration should be given to using EOs that possess both acaricidal activity and oviposition inhibition. Furthermore, the combination of highly toxic and oviposition-inhibiting EOs can be applied to minimize the population size of existing mites and reduce the number of future generations, thus reducing the frequency of pesticide use.

## 4. Materials and Methods

### 4.1. Plants, Pest Mites, and Essential Oils

Plants: Pea seeds (Sichuan Kexi, Sichuan, China) were planted in a white plastic pot with dimensions of 29 cm × 21.5 cm × 7.5 cm, using a mixed substrate of peat, vermiculite, and perlite. The cultivation took place in an incubator with a temperature of (25 ± 2) °C, humidity of (60 ± 10)%, and a light cycle of L:D = 16 h:8 h.

Pest mites: *T. urticae*, collected from strawberry plants in a greenhouse located in Hangzhou, China, were subsequently transferred to clean pea seedlings to establish a population. For the experiment, female adult mites with similar body size, vibrant color, and strong mobility were selected.

Essential oils: The EOs were purchased from Guangzhou Fengya Pharmaceutical Technology Co., Ltd. (Foshan, China) and Guangzhou Hongli Biotechnology Co., Ltd. (Guangzhou, China). The information of EOs is shown in Table 3, confirming that all EOs were 100% concentrated.

### 4.2. Toxicity Determination of EOs against T. urticae

Following the method described by Subaharan et al., the EOs, added to a 0.1% Tween-80 solution (Shanghai Macklin Biochemical Technology, Shanghai, China), were subjected to 15 min of ultrasonication to emulsify the EOs and produce an EO microemulsion with a concentration of 4 μL/mL [44].

Tender leaves were selected from pea seedlings, which were immersed in the EO solution for 10 s and then air-dried naturally. The leaves were placed with their undersides facing up on 1% agar plates. Twenty female *T. urticae* of consistent age were selected and inoculated onto each leaf agar-plate. The prepared agar plates were transferred to the incubator. The leaf agar-plates were examined under a microscope at 6 h, 12 h, 24 h, 48 h, and 72 h after mite inoculation, and the mortality of female mites in each treatment group was observed and recorded. A 0.1% Tween-80 solution was used as the control, and each treatment was replicated three times.

### 4.3. Determination of Oviposition Inhibitory Effects of EOs on T. urticae

The leaf-agar plates treated with EOs were prepared according to the aforementioned method. Each leaf-agar plate was inoculated with 20 female mites and placed in the incubator. The oviposition was observed and recorded at 12 h, 24 h, 48 h, and 72 h after mite inoculation. A 0.1% Tween-80 solution was used as the control, and each treatment was replicated three times.

### 4.4. Data Analysis

Following the methodology described by Zhu et al. (2023), statistical analysis was conducted using Polo-Plus software Version: 1.0 to calculate LC_50_, 95% confidence limits, and toxicity regression equation [23]. GraphPad Prism 9 was employed for one-Way ANOVA (analysis of variance), Dunnett’s multiple comparisons, and graphical visualization. 

The treatment groups were compared based on their oviposition inhibition index and oviposition inhibition rate to evaluate the inhibitory effect of 38 EOs on *T. urticae* oviposition. Oviposition inhibition index = (eggs of control − eggs of treatments)/(eggs of control + eggs of treatments); oviposition inhibition rate = (eggs of control − eggs of treatments)/(eggs of control).

GraphPad Prism 9 was then used to plot regression lines using simple linear regression and simple logistic regression to analyze the relationship between oviposition inhibition rate and mortality rate.

## 5. Conclusions

This study determined the toxicity and oviposition inhibition of EOs against *T. urticae*. The results demonstrate that the mortality rate of *T. urticae* increases with prolonged exposure to EOs. Thirteen EOs exhibited acaricidal activity, and the mortality rate of *T. urticae* was significantly higher compared to the control group. The toxicity level from high to low was as follows: lemon, sage, frankincense, palmarosa, patchouli, lemongrass, oregano, jasmine, peppermint, clove, tea tree, geranium, and nerol. The LC_50_ values of the top four EO treatments were below 5 μL/mL. The oviposition inhibition rates of lemon, sage, frankincense, clove, lemongrass, oregano, palmarosa, myrrh, black pepper, rose geranium, and geranium EOs all exceeded 50%. Although black pepper, clove, myrrh, and oregano EOs exhibited relatively low toxicity against *T. urticae* at this concentration, their oviposition inhibition activity was high. Additionally, this study found that certain EOs can stimulate oviposition in *T. urticae*, suggesting the need for further research on the mechanisms of action of these EOs. However, in this study, only the leaf-dipping bioassay was used to treat *T. urticae*. The fumigation and repellent activity of essential oil against *T. urticae* can be studied in the future, and the effects of different treatment methods can be compared to explore more convenient and efficient application measures of plant essential oil to control *T. urticae*. 

## Figures and Tables

**Table 1:** The LC_50_ values and toxicity regression equations of the tested plant essential oils against *T. urticae* at different time intervals.

EOs	6 h		12 h		24 h		48 h		72 h	
	LC_50_ (μL/mL) 95% CL	Regression Equation	LC_50_ (μL/mL) 95% CL	Regression Equation	LC_50_ (μL/mL) 95% CL	Regression Equation	LC_50_ (μL/mL) 95% CL	Regression Equation	LC_50_ (μL/mL) 95% CL	Regression Equation
frankincense	25.064 13.012~817.163	y = 1.0906ln(x) + 1.4438	14.994 8.886~163.901	y = 1.0761ln(x) + 2.2349	9.435 6.607~31.513	y = 1.3868ln(x) + 1.9405	6.067 4.270~12.090	y = 1.8452ln(x) + 1.6412	4.611 3.206~7.105	y = 2.3934ln(x) + 1.073
lemongrass	-	-	12.225 8.170~36.978	y = 1.2095ln(x) + 2.1351	8.205 5.708~23.423	y = 1.377ln(x) + 2.1884	7.142 5.116~15.579	y = 1.4857ln(x) + 2.118	6.196 4.973~8.538	y = 1.4856ln(x) + 2.2852
lemon	13.036 8.890~26.508	y = 1.1005ln(x) + 2.2964	6.203 4.164~10.083	y = 1.3344ln(x) + 2.4365	3.539 2.332~5.142	y = 1.1263ln(x) + 3.3387	2.851 1.562~4.470	y = 1.2371ln(x) + 3.4333	2.310 1.117~3.692	y = 1.1837ln(x) + 3.841
patchouli	-	-	15.721 9.831~92.783	y = 1.0997ln(x) + 1.8758	6.795 5.259~10.178	y = 1.6683ln(x) + 1.6055	4.853 4.482~5.320	y = 2.657ln(x) + 1.2572	4.202 3.810~4.604	y = 2.7609ln(x) + 1.3018
palmarosa	82.958 33.573~2565.803	y = 0.7385ln(x) + 1.7545	18.586 13.787~36.748	y = 1.0735ln(x) + 1.5465	11.387 8.762~16.731	y = 1.369ln(x) + 1.412	7.865 6.057~10.216	y = 1.8096ln(x) + 1.3555	6.887 5.193~8.551	y = 1.7287ln(x) + 2.0848
sage	11.320 9.945~13.176	y = 1.8253ln(x) + 0.4772	6.804 3.994~14.357	y = 1.4205ln(x) + 2.0342	6.588 4.161~9.037	y = 1.4949ln(x) + 2.3663	4.116 2.710~5.743	y = 1.9369ln(x) + 2.1153	3.684 2.059~5.728	y = 1.904ln(x) + 2.4796
oregano	37.022 21.843~143.552	y = 0.8207ln(x) + 1.895	12.358 9.672~17.793	y = 1.2328ln(x) + 1.9051	9.733 7.055~15.860	y = 1.3429ln(x) + 2.2048	9.551 6.490~14.821	y = 1.2911ln(x) + 2.527	9.123 6.245~13.072	y = 1.4264ln(x) + 2.2606

**Table 2:** Oviposition inhibition indices of *T. urticae* treated with 4 μL/mL plant essential oils at different time intervals.

EOs	12 h	24 h	48 h	72 h
black pepper	0.33 ± 0.05	0.51 ± 0.06	0.56 ± 0.06	0.59 ± 0.08
bergamot	0.29 ± 0.45	0.34 ± 0.32	0.32 ± 0.25	0.29 ± 0.25
basil	0.26 ± 0.03	0.32 ± 0.13	0.27 ± 0.22	0.26 ± 0.26
cinnamon	0.00 ± 0.24	−0.06 ± 0.21	0.00 ± 0.25	−0.07 ± 0.26
clove	0.59 ± 0.14	0.60 ± 0.13	0.69 ± 0.17	0.73 ± 0.12
cypress	0.24 ± 0.34	0.06 ± 0.18	0.06 ± 0.12	0.00 ± 0.11
chamomile	0.13 ± 0.10	−0.04 ± 0.12	−0.01 ± 0.14	0.03 ± 0.10
citrus	0.25 ± 0.18	0.29 ± 0.09	0.21 ± 0.18	0.20 ± 0.09
eucalyptus	0.03 ± 0.14	0.02 ± 0.04	0.06 ± 0.09	0.15 ± 0.12
frankincense	0.78 ± 0.21	0.63 ± 0.32	0.76 ± 0.21	0.77 ± 0.20
geranium	0.11 ± 0.38	0.21 ± 0.36	0.42 ± 0.30	0.36 ± 0.15
ginger	0.27 ± 0.43	0.36 ± 0.41	0.15 ± 0.17	0.19 ± 0.24
grapefruit	−0.25 ± 0.05	−0.26 ± 0.10	−0.22 ± 0.23	−0.13 ± 0.26
juniper berry	−0.03 ± 0.23	−0.11 ± 0.00	0.13 ± 0.05	0.23 ± 0.00
jasmine	−0.19 ± 0.36	−0.34 ± 0.08	−0.27 ± 0.06	−0.31 ± 0.05
lavender	0.08 ± 0.26	−0.19 ± 0.08	−0.10 ± 0.14	−0.05 ± 0.09
lemongrass	0.80 ± 0.20	0.72 ± 0.25	0.78 ± 0.19	0.75 ± 0.20
lemon	1.00 ± 0.00	1.00 ± 0.00	1.00 ± 0.00	0.98 ± 0.03
myrrh	0.32 ± 0.36	0.45 ± 0.31	0.56 ± 0.24	0.61 ± 0.15
melissa	−0.24 ± 0.10	−0.20 ± 0.08	−0.12 ± 0.08	−0.18 ± 0.05
niaouli	0.17 ± 0.26	0.13 ± 0.26	0.00 ± 0.28	0.01 ± 0.24
neroli	0.01 ± 0.38	0.09 ± 0.44	0.05 ± 0.43	0.07 ± 0.42
nutmeg	0.11 ± 0.12	0.07 ± 0.02	0.16 ± 0.07	0.13 ± 0.02
oregano	0.58 ± 0.14	0.69 ± 0.09	0.72 ± 0.11	0.73 ± 0.10
peppermint	0.06 ± 0.23	0.02 ± 0.15	0.05 ± 0.12	0.15 ± 0.11
pine needles	−0.15 ± 0.21	−0.23 ± 0.14	−0.20 ± 0.10	−0.23 ± 0.11
patchouli	0.59 ± 0.08	0.64 ± 0.11	0.59 ± 0.12	0.66 ± 0.11
palmarosa	0.40 ± 0.20	0.48 ± 0.14	0.55 ± 0.13	0.54 ± 0.14
ravansara	−0.10 ± 0.12	−0.17 ± 0.04	−0.08 ± 0.09	−0.02 ± 0.10
rose	0.26 ± 0.16	0.29 ± 0.12	0.26 ± 0.29	0.28 ± 0.25
rosemary	−0.02 ± 0.14	−0.01 ± 0.24	0.01 ± 0.22	0.02 ± 0.11
sage	0.77 ± 0.08	0.82 ± 0.07	0.87 ± 0.05	0.86 ± 0.09
sweet orange	−0.18 ± 0.31	−0.23 ± 0.43	−0.23 ± 0.24	−0.11 ± 0.26
tea tree	−0.24 ± 0.25	−0.12 ± 0.34	−0.04 ± 0.39	0.03 ± 0.35
thyme	−0.21 ± 0.25	−0.30 ± 0.17	−0.27 ± 0.12	−0.20 ± 0.13
vanilla	−0.33 ± 0.13	−0.28 ± 0.30	−0.29 ± 0.34	−0.21 ± 0.37
vetiver	0.53 ± 0.35	0.61 ± 0.27	0.22 ± 0.06	0.21 ± 0.09
ylang	−0.20 ± 0.05	−0.03 ± 0.06	0.01 ± 0.13	−0.04 ± 0.11

**Table 3:** Names and origins of essential oils.

EOs	Species	Family	EOs	Species	Family
black pepper	*Piper nigrum*	Piperaceae	melissa	*Melissa officinalis*	Lamiaceae
bergamot	*Citrus medica* ‘Fingered’	Rutaceae	niaouli	*Melaleuca viridiflora*	Myrtaceae
basil	*Ocimum basilicum*	Lamiaceae	neroli	*Citrus × aurantium*	Rutaceae
cinnamon	*Cinnamomum cassia*	Lauraceae	nutmeg	*Alpinia katsumadai*	Zingiberaceae
clove	*Syringa oblata*	Oleaceae	oregano	*Origanum vulgare*	Lamiaceae
cypress	*Cupressus sempervirens*	Cupressaceae	peppermint	*Mentha canadensis*	Lamiaceae
chamomile	*Matricaria chamomilla*	Asteraceae	pine needles	*Pinus*	Pinaceae
citrus	*Citrus reticulata*	Rutaceae	patchouli	*Pogostemon cablin*	Lamiaceae
eucalyptus	*Eucalyptus robusta*	Myrtaceae	palmarosa	*Cymbopogon martini*	Poaceae
frankincense	*Boswellia carteri*	Burseraceae	ravansara	*Ravensara aromatica*	Lauraceae
geranium	*Pelargonium hortorum*	Geraniaceae	rose	*Rosa rugosa*	Rosaceae
ginger	*Zingiber officinale*	Zingiberaceae	rosemary	*Rosmarinus officinalis*	Lamiaceae
grapefruit	*Citrus × aurantium*	Rutaceae	sage	*Salvia japonica*	Lamiaceae
juniper berry	*Juniperus rigida*	Cupressaceae	sweet orange	*Citrus sinensis*	Rutaceae
jasmine	*Jasminum sambac*	Oleaceae	tea tree	*Camellia sinensis*	Theaceae
lavender	*Lavandula angustifolia*	Lamiaceae	thyme	*Thymus mongolicus*	Lamiaceae
lemongrass	*Cymbopogon citratus*	Poaceae	vanilla	*Vanilla planifolia*	Orchidaceae
lemon	*Citrus × limon*	Rutaceae	vetiver	*Chrysopogon zizanioides*	Poaceae
myrrh	*Eucalyptus robusta*	Burseraceae	ylang	*Cananga odorata*	Annonaceae

## Data Availability

Data available on request from the authors.
